# Ethyl 2-methyl-5-oxo-4-(3,4,5-trimeth­oxy­phen­yl)-1,4,5,6,7,8-hexa­hydro­quinoline-3-carboxyl­ate

**DOI:** 10.1107/S1600536810030333

**Published:** 2010-08-11

**Authors:** S. Natarajan, P. Indumathi, B. Palakshi Reddy, V. Vijayakumar, P. L. Nilantha Lakshman

**Affiliations:** a Department of Physics, Madurai Kamaraj University, Madurai 625 021, India; b Organic Chemistry Division, School of Advanced Sciences, VIT University, Vellore 632 104, India; c Department of Food Science and Technology, Faculty of Agriculture, University of Ruhuna, Mapalana, Kamburupitiya (81100), Sri Lanka

## Abstract

In the mol­ecular structure of the title compound, C_22_H_27_NO_6_, the dihydro­pyridine ring adopts a flattened boat conformation while the cyclo­hexenone ring is in an envelope conformation. In the crystal, mol­ecules stack parallel to the crystallographic *a* axis linked by inter­molecular N—H⋯O and C—H⋯O hydrogen bonds.

## Related literature

For general background to the biological activity of quinoline derivatives, see: Baba (1997 ▶); Baba *et al.* (1997 ▶,1998 ▶); Davies *et al.* (2005 ▶); Rose & Draeger *et al.* (1992 ▶); Warrior *et al.* (2005 ▶).
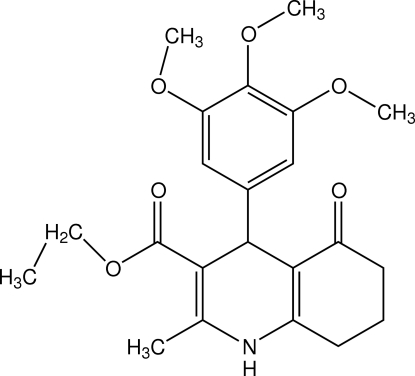


## Experimental

### 

#### Crystal data

C_22_H_27_NO_6_
*M* *_r_* = 401.44
Triclinic, 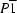

*a* = 7.512 (2) Å
*b* = 10.402 (1) Å
*c* = 14.568 (3) Å
α = 109.77 (3)°
β = 95.42 (1)°
γ = 104.41 (2)°
*V* = 1017.4 (4) Å^3^
*Z* = 2
Mo *K*α radiation
μ = 0.10 mm^−1^
*T* = 294 K
0.26 × 0.24 × 0.21 mm

#### Data collection

Nonius MACH3 diffractometer
Absorption correction: ψ scan (North *et al.*, 1968 ▶) *T*_min_ = 0.976, *T*_max_ = 0.980
4471 measured reflections
3574 independent reflections
2653 reflections with *I* > 2σ(*I*)
*R*_int_ = 0.013
3 standard reflections every 60 min  intensity decay: none

#### Refinement

*R*[*F* ^2^ > 2σ(*F* ^2^)] = 0.043
*wR*(*F* ^2^) = 0.129
*S* = 1.03
3574 reflections
268 parameters
H atoms treated by a mixture of independent and constrained refinement
Δρ_max_ = 0.23 e Å^−3^
Δρ_min_ = −0.18 e Å^−3^

### 

Data collection: *CAD-4 EXPRESS* (Enraf–Nonius, 1994 ▶); cell refinement: *CAD-4 EXPRESS*; data reduction: *XCAD4* (Harms & Wocadlo, 1996 ▶); program(s) used to solve structure: *SHELXS97* (Sheldrick, 2008 ▶); program(s) used to refine structure: *SHELXL97* (Sheldrick, 2008 ▶); molecular graphics: *PLATON* (Spek, 2009 ▶); software used to prepare material for publication: *SHELXL97*.

## Supplementary Material

Crystal structure: contains datablocks global, I. DOI: 10.1107/S1600536810030333/jh2183sup1.cif

Structure factors: contains datablocks I. DOI: 10.1107/S1600536810030333/jh2183Isup2.hkl

Additional supplementary materials:  crystallographic information; 3D view; checkCIF report

## Figures and Tables

**Table 1:** Hydrogen-bond geometry (Å, °)

*D*—H⋯*A*	*D*—H	H⋯*A*	*D*⋯*A*	*D*—H⋯*A*
N1—H1*N*⋯O1^i^	0.83 (3)	2.21 (3)	2.995 (2)	160 (3)
C2—H2*B*⋯O4^ii^	0.97	2.55	3.340 (3)	138
C10—H10*B*⋯O1^i^	0.96	2.59	3.429 (3)	146

Symmetry codes: (i) 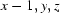; (ii) 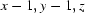.

**Fig. 1.**
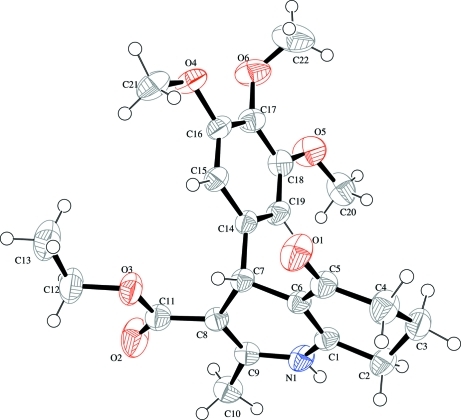
The molecular structure of (I), showing 50% probability displacement ellipsoids and the atom-numbering scheme.

**Fig. 2.**
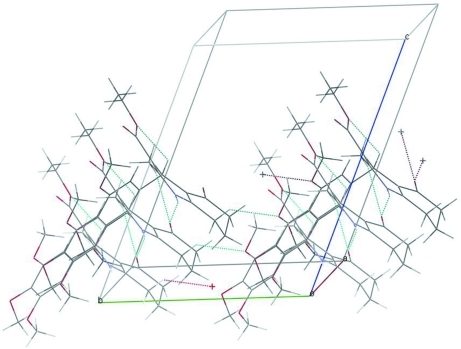
The packing diagram showing the sheets of hydrogen bonds along the a axis

## supplementary crystallographic information

### Comment

Some derivatives of quinoline are naturally occurring alkaloids and are very attractive for their various bioactivities. For example, they have calcium modulatory properties (Rose &Draeger, 1992), antibacterial activity (Davies *et al.*, 2005),fungicidal activity (Warrior *et al.*, 2005) and selective inhibitor of human immunodeficiency virus type I (HIV-1) transcription (Baba, 1997; Baba *et al.*,1997,1998) *etc*. Due to these significant biological activities, the structure of a quinoline derivative, ethyl 2-methyl-5-oxo -4-(3,4,5-trimethoxyphenyl)-1,4,5,6,7,8-hexahydroquinoline-3-carboxylate is elucidated and reported.

The dihydropyridine ring of the title molecule adopts a flattened boat conformation. The cyclohexenone ring is in an envelope conformation with atom C3 at the flap. The 3,4,5-trimethoxyphenyl ring and the plane of the dihydropyridine ring (N1/C1/C6/C7/C8/C9) are nearly perpendicular to each other, with a dihedral angle of 89.33 (4)°. In the crystal structure, molecules are linked into a sheet (Fig.2) parallel to the *a* axis by N—H···O and C—H···O intra and intermolecular hydrogen bonds (Table 1). Further, it is observed that these sheets are assembled through centrosymmetrically related pairs of molecules by C2—H2B···O4 inter molecular hydrogen bond and weak interactions, which stabilize the structure.

### Experimental

3,4,5-trimethoxy benzaldehyde (10 mmol), 1,3-cyclohexanedione (10 mmol) and ethyl acetoacetate (10 mmol) were mixed along with 20 ml of ethanol. Ammonium acetate (10 mmol) was added to the mixture and refluxed on water bath for about 1 h. The progress of the reaction was monitored by TLC. After confirming that the reaction got completed, the reaction mixture was allowed to cool to room temperature and left aside for a day. Solid crystals started to grow from the mother liquor. It was filtered and washed with diethyl ether to ensure pure crystals [yield: 60%, m.p. 576–578 K].

### Refinement

H atoms were placed at calculated positions and allowed to ride on their carrier atoms with N—H = 0.83 Å, C—H = 0.93–0.97 Å, and *U*_iso_ = 1.2*U*_eq_(C) for CH_2_ and CH groups and *U*_iso_ = 1.5*U*_eq_(C) for CH_3_ group.

### Crystal data

**Table:**

C_22_H_27_NO_6_	*Z* = 2
*M**_r_* = 401.44	*F*(000) = 428
Triclinic, *P*1	*D*_x_ = 1.310 Mg m^−^^3^
Hall symbol: -P 1	Mo *K*α radiation, λ = 0.71073 Å
*a* = 7.512 (2) Å	Cell parameters from 25 reflections
*b* = 10.402 (1) Å	θ = 2–25°
*c* = 14.568 (3) Å	µ = 0.10 mm^−^^1^
α = 109.77 (3)°	*T* = 294 K
β = 95.42 (1)°	Block, colourless
γ = 104.41 (2)°	0.26 × 0.24 × 0.21 mm
*V* = 1017.4 (4) Å^3^	

### Data collection

**Table:**

Nonius MACH3 diffractometer	2653 reflections with *I* > 2σ(*I*)
Radiation source: fine-focus sealed tube	*R*_int_ = 0.013
graphite	θ_max_ = 25.0°, θ_min_ = 2.1°
ω–2θ scans	*h* = −1→8
Absorption correction: ψ scan (North *et al.*, 1968)	*k* = −12→12
*T*_min_ = 0.976, *T*_max_ = 0.980	*l* = −17→17
4471 measured reflections	3 standard reflections every 60 min
3574 independent reflections	intensity decay: none

### Refinement

**Table:**

Refinement on *F*^2^	Secondary atom site location: difference Fourier map
Least-squares matrix: full	Hydrogen site location: inferred from neighbouring sites
*R*[*F*^2^ > 2σ(*F*^2^)] = 0.043	H atoms treated by a mixture of independent and constrained refinement
*wR*(*F*^2^) = 0.129	*w* = 1/[σ^2^(*F*_o_^2^) + (0.0609*P*)^2^ + 0.3979*P*] where *P* = (*F*_o_^2^ + 2*F*_c_^2^)/3
*S* = 1.03	(Δ/σ)_max_ < 0.001
3574 reflections	Δρ_max_ = 0.23 e Å^−^^3^
268 parameters	Δρ_min_ = −0.18 e Å^−^^3^
0 restraints	Extinction correction: *SHELXL97* (Sheldrick, 2008), Fc^*^=kFc[1+0.001xFc^2^λ^3^/sin(2θ)]^-1/4^
Primary atom site location: structure-invariant direct methods	Extinction coefficient: 0.013 (3)

### Special details

**Table:**

Geometry. All e.s.d.'s (except the e.s.d. in the dihedral angle between two l.s. planes) are estimated using the full covariance matrix. The cell e.s.d.'s are taken into account individually in the estimation of e.s.d.'s in distances, angles and torsion angles; correlations between e.s.d.'s in cell parameters are only used when they are defined by crystal symmetry. An approximate (isotropic) treatment of cell e.s.d.'s is used for estimating e.s.d.'s involving l.s. planes.
Refinement. Refinement of *F*^2^ against ALL reflections. The weighted *R*-factor *wR* and goodness of fit *S* are based on *F*^2^, conventional *R*-factors *R* are based on *F*, with *F* set to zero for negative *F*^2^. The threshold expression of *F*^2^ > σ(*F*^2^) is used only for calculating *R*-factors(gt) *etc*. and is not relevant to the choice of reflections for refinement. *R*-factors based on *F*^2^ are statistically about twice as large as those based on *F*, and *R*- factors based on ALL data will be even larger.

### Fractional atomic coordinates and isotropic or equivalent isotropic displacement parameters (Å^2^)

**Table:**

	*x*	*y*	*z*	*U*_iso_*/*U*_eq_	
C22	0.4600 (4)	0.2952 (4)	0.0056 (2)	0.0841 (9)	
H22A	0.4922	0.3586	−0.0293	0.101*	
H22B	0.3534	0.2153	−0.0339	0.101*	
H22C	0.5642	0.2613	0.0178	0.101*	
H1N	−0.286 (4)	−0.135 (3)	0.2897 (18)	0.061 (7)*	
O4	0.68460 (19)	0.35699 (15)	0.22606 (11)	0.0519 (4)	
O3	0.36747 (19)	0.26111 (15)	0.50993 (10)	0.0523 (4)	
N1	−0.1712 (2)	−0.09684 (18)	0.30488 (13)	0.0405 (4)	
O1	0.41288 (19)	−0.16053 (16)	0.25103 (12)	0.0588 (4)	
C6	0.1238 (2)	−0.11864 (19)	0.27165 (13)	0.0323 (4)	
C1	−0.0644 (2)	−0.17812 (19)	0.25700 (13)	0.0347 (4)	
C8	0.0925 (2)	0.09809 (19)	0.40306 (13)	0.0346 (4)	
C9	−0.0944 (2)	0.0320 (2)	0.38398 (14)	0.0356 (4)	
C15	0.4536 (2)	0.20151 (19)	0.27900 (14)	0.0371 (4)	
H15	0.5452	0.1991	0.3256	0.044*	
O2	0.0990 (2)	0.30115 (19)	0.54511 (14)	0.0765 (6)	
C5	0.2404 (3)	−0.2085 (2)	0.23209 (15)	0.0407 (5)	
C16	0.5036 (3)	0.28065 (19)	0.22121 (15)	0.0388 (4)	
O6	0.4166 (2)	0.36880 (18)	0.09651 (13)	0.0659 (5)	
C19	0.1316 (3)	0.1295 (2)	0.19881 (14)	0.0393 (4)	
H19	0.0068	0.0788	0.1914	0.047*	
C7	0.2165 (2)	0.03861 (18)	0.33288 (13)	0.0330 (4)	
H7	0.3332	0.0471	0.3740	0.040*	
C14	0.2679 (2)	0.12545 (18)	0.26814 (13)	0.0332 (4)	
O5	0.0588 (2)	0.21801 (19)	0.06860 (13)	0.0656 (5)	
C3	−0.0564 (3)	−0.3963 (2)	0.12134 (17)	0.0557 (6)	
H3A	−0.0602	−0.3578	0.0694	0.067*	
H3B	−0.1143	−0.4991	0.0910	0.067*	
C18	0.1810 (3)	0.2088 (2)	0.14062 (15)	0.0430 (5)	
C17	0.3684 (3)	0.2858 (2)	0.15175 (15)	0.0438 (5)	
C11	0.1788 (3)	0.2281 (2)	0.49145 (15)	0.0446 (5)	
C2	−0.1646 (3)	−0.3315 (2)	0.19699 (16)	0.0448 (5)	
H2A	−0.2869	−0.3398	0.1631	0.054*	
H2B	−0.1832	−0.3840	0.2408	0.054*	
C10	−0.2342 (3)	0.0813 (2)	0.44419 (16)	0.0479 (5)	
H10A	−0.1986	0.0885	0.5112	0.058*	
H10B	−0.3565	0.0135	0.4152	0.058*	
H10C	−0.2366	0.1733	0.4445	0.058*	
C4	0.1457 (3)	−0.3643 (2)	0.16960 (18)	0.0539 (6)	
H4A	0.1496	−0.4198	0.2111	0.065*	
H4B	0.2156	−0.3952	0.1179	0.065*	
C20	−0.1304 (3)	0.1336 (3)	0.04654 (18)	0.0630 (7)	
H20A	−0.1993	0.1503	−0.0050	0.076*	
H20B	−0.1835	0.1588	0.1051	0.076*	
H20C	−0.1369	0.0342	0.0244	0.076*	
C21	0.8277 (3)	0.3578 (3)	0.29756 (19)	0.0573 (6)	
H21A	0.9466	0.4146	0.2935	0.069*	
H21B	0.8305	0.2616	0.2845	0.069*	
H21C	0.8025	0.3978	0.3630	0.069*	
C13	0.6642 (3)	0.4322 (3)	0.5930 (2)	0.0768 (8)	
H13A	0.7287	0.5156	0.6512	0.092*	
H13B	0.6768	0.4544	0.5347	0.092*	
H13C	0.7174	0.3564	0.5904	0.092*	
C12	0.4662 (3)	0.3867 (3)	0.59723 (18)	0.0667 (7)	
H12A	0.4530	0.3652	0.6565	0.080*	
H12B	0.4124	0.4635	0.6007	0.080*	

### Atomic displacement parameters (Å^2^)

**Table:**

	*U*^11^	*U*^22^	*U*^33^	*U*^12^	*U*^13^	*U*^23^
C22	0.0715 (18)	0.133 (3)	0.080 (2)	0.0367 (18)	0.0272 (15)	0.072 (2)
O4	0.0339 (8)	0.0517 (9)	0.0690 (10)	0.0004 (6)	0.0116 (7)	0.0298 (8)
O3	0.0343 (8)	0.0524 (9)	0.0480 (8)	0.0054 (6)	0.0031 (6)	−0.0016 (7)
N1	0.0211 (8)	0.0445 (9)	0.0519 (10)	0.0076 (7)	0.0058 (7)	0.0150 (8)
O1	0.0272 (8)	0.0560 (9)	0.0804 (11)	0.0141 (7)	0.0118 (7)	0.0085 (8)
C6	0.0257 (9)	0.0357 (10)	0.0354 (9)	0.0083 (7)	0.0064 (7)	0.0137 (8)
C1	0.0276 (9)	0.0381 (10)	0.0394 (10)	0.0079 (8)	0.0057 (8)	0.0172 (8)
C8	0.0294 (9)	0.0378 (10)	0.0375 (10)	0.0117 (8)	0.0073 (8)	0.0139 (8)
C9	0.0310 (10)	0.0411 (10)	0.0407 (10)	0.0154 (8)	0.0094 (8)	0.0189 (8)
C15	0.0280 (9)	0.0374 (10)	0.0428 (11)	0.0072 (8)	0.0046 (8)	0.0139 (8)
O2	0.0504 (10)	0.0693 (11)	0.0758 (12)	0.0162 (8)	0.0164 (9)	−0.0140 (9)
C5	0.0321 (10)	0.0433 (11)	0.0455 (11)	0.0111 (8)	0.0085 (8)	0.0152 (9)
C16	0.0322 (10)	0.0323 (10)	0.0487 (11)	0.0065 (8)	0.0120 (8)	0.0128 (8)
O6	0.0674 (11)	0.0684 (11)	0.0800 (12)	0.0172 (9)	0.0201 (9)	0.0508 (10)
C19	0.0279 (9)	0.0405 (10)	0.0452 (11)	0.0065 (8)	0.0061 (8)	0.0138 (9)
C7	0.0230 (8)	0.0367 (10)	0.0379 (10)	0.0082 (7)	0.0054 (7)	0.0129 (8)
C14	0.0278 (9)	0.0311 (9)	0.0370 (10)	0.0084 (7)	0.0077 (7)	0.0082 (8)
O5	0.0493 (10)	0.0834 (12)	0.0730 (11)	0.0173 (8)	−0.0018 (8)	0.0458 (10)
C3	0.0474 (13)	0.0447 (12)	0.0554 (13)	0.0020 (10)	0.0087 (10)	0.0035 (10)
C18	0.0408 (11)	0.0454 (11)	0.0438 (11)	0.0160 (9)	0.0042 (9)	0.0167 (9)
C17	0.0456 (12)	0.0406 (11)	0.0496 (12)	0.0129 (9)	0.0128 (9)	0.0215 (9)
C11	0.0380 (11)	0.0462 (11)	0.0463 (11)	0.0114 (9)	0.0111 (9)	0.0133 (9)
C2	0.0322 (10)	0.0419 (11)	0.0521 (12)	0.0030 (8)	0.0034 (9)	0.0145 (9)
C10	0.0340 (11)	0.0593 (13)	0.0570 (13)	0.0217 (9)	0.0163 (9)	0.0223 (10)
C4	0.0470 (12)	0.0435 (12)	0.0636 (14)	0.0163 (10)	0.0146 (11)	0.0080 (10)
C20	0.0466 (13)	0.0887 (18)	0.0515 (13)	0.0309 (13)	0.0002 (10)	0.0186 (13)
C21	0.0307 (11)	0.0586 (14)	0.0789 (16)	0.0055 (10)	0.0112 (11)	0.0268 (12)
C13	0.0482 (14)	0.0747 (17)	0.0697 (17)	0.0042 (12)	−0.0008 (12)	−0.0059 (14)
C12	0.0475 (13)	0.0650 (15)	0.0528 (14)	0.0008 (11)	0.0051 (11)	−0.0078 (12)

### Geometric parameters (Å, °)

**Table:**

C22—O6	1.402 (3)	C19—H19	0.9300
C22—H22A	0.9600	C7—C14	1.525 (2)
C22—H22B	0.9600	C7—H7	0.9800
C22—H22C	0.9600	O5—C18	1.372 (2)
O4—C16	1.375 (2)	O5—C20	1.413 (3)
O4—C21	1.421 (3)	C3—C2	1.506 (3)
O3—C11	1.350 (2)	C3—C4	1.516 (3)
O3—C12	1.446 (3)	C3—H3A	0.9700
N1—C1	1.374 (2)	C3—H3B	0.9700
N1—C9	1.379 (3)	C18—C17	1.399 (3)
N1—H1N	0.83 (3)	C2—H2A	0.9700
O1—C5	1.234 (2)	C2—H2B	0.9700
C6—C1	1.358 (2)	C10—H10A	0.9600
C6—C5	1.453 (3)	C10—H10B	0.9600
C6—C7	1.512 (3)	C10—H10C	0.9600
C1—C2	1.486 (3)	C4—H4A	0.9700
C8—C9	1.355 (2)	C4—H4B	0.9700
C8—C11	1.461 (3)	C20—H20A	0.9600
C8—C7	1.528 (2)	C20—H20B	0.9600
C9—C10	1.508 (3)	C20—H20C	0.9600
C15—C16	1.379 (3)	C21—H21A	0.9600
C15—C14	1.386 (2)	C21—H21B	0.9600
C15—H15	0.9300	C21—H21C	0.9600
O2—C11	1.209 (2)	C13—C12	1.458 (3)
C5—C4	1.505 (3)	C13—H13A	0.9600
C16—C17	1.389 (3)	C13—H13B	0.9600
O6—C17	1.376 (2)	C13—H13C	0.9600
C19—C18	1.384 (3)	C12—H12A	0.9700
C19—C14	1.387 (3)	C12—H12B	0.9700
			
O6—C22—H22A	109.5	O5—C18—C19	125.06 (18)
O6—C22—H22B	109.5	O5—C18—C17	114.73 (18)
H22A—C22—H22B	109.5	C19—C18—C17	120.20 (18)
O6—C22—H22C	109.5	O6—C17—C16	120.62 (18)
H22A—C22—H22C	109.5	O6—C17—C18	120.21 (18)
H22B—C22—H22C	109.5	C16—C17—C18	119.13 (18)
C16—O4—C21	117.61 (16)	O2—C11—O3	121.13 (19)
C11—O3—C12	116.21 (17)	O2—C11—C8	126.93 (19)
C1—N1—C9	122.70 (16)	O3—C11—C8	111.93 (16)
C1—N1—H1N	116.1 (17)	C1—C2—C3	111.44 (16)
C9—N1—H1N	120.6 (17)	C1—C2—H2A	109.3
C1—C6—C5	119.71 (16)	C3—C2—H2A	109.3
C1—C6—C7	121.31 (16)	C1—C2—H2B	109.3
C5—C6—C7	118.90 (15)	C3—C2—H2B	109.3
C6—C1—N1	119.52 (17)	H2A—C2—H2B	108.0
C6—C1—C2	124.04 (17)	C9—C10—H10A	109.5
N1—C1—C2	116.30 (16)	C9—C10—H10B	109.5
C9—C8—C11	120.34 (17)	H10A—C10—H10B	109.5
C9—C8—C7	120.86 (17)	C9—C10—H10C	109.5
C11—C8—C7	118.80 (16)	H10A—C10—H10C	109.5
C8—C9—N1	119.58 (17)	H10B—C10—H10C	109.5
C8—C9—C10	126.59 (18)	C5—C4—C3	113.84 (18)
N1—C9—C10	113.77 (16)	C5—C4—H4A	108.8
C16—C15—C14	120.44 (18)	C3—C4—H4A	108.8
C16—C15—H15	119.8	C5—C4—H4B	108.8
C14—C15—H15	119.8	C3—C4—H4B	108.8
O1—C5—C6	121.43 (18)	H4A—C4—H4B	107.7
O1—C5—C4	120.26 (18)	O5—C20—H20A	109.5
C6—C5—C4	118.29 (16)	O5—C20—H20B	109.5
O4—C16—C15	124.14 (18)	H20A—C20—H20B	109.5
O4—C16—C17	115.44 (17)	O5—C20—H20C	109.5
C15—C16—C17	120.42 (17)	H20A—C20—H20C	109.5
C17—O6—C22	113.52 (19)	H20B—C20—H20C	109.5
C18—C19—C14	120.14 (17)	O4—C21—H21A	109.5
C18—C19—H19	119.9	O4—C21—H21B	109.5
C14—C19—H19	119.9	H21A—C21—H21B	109.5
C6—C7—C14	112.18 (15)	O4—C21—H21C	109.5
C6—C7—C8	110.40 (14)	H21A—C21—H21C	109.5
C14—C7—C8	111.39 (14)	H21B—C21—H21C	109.5
C6—C7—H7	107.6	C12—C13—H13A	109.5
C14—C7—H7	107.6	C12—C13—H13B	109.5
C8—C7—H7	107.6	H13A—C13—H13B	109.5
C15—C14—C19	119.66 (17)	C12—C13—H13C	109.5
C15—C14—C7	119.36 (16)	H13A—C13—H13C	109.5
C19—C14—C7	120.98 (16)	H13B—C13—H13C	109.5
C18—O5—C20	118.35 (18)	O3—C12—C13	110.0 (2)
C2—C3—C4	110.79 (18)	O3—C12—H12A	109.7
C2—C3—H3A	109.5	C13—C12—H12A	109.7
C4—C3—H3A	109.5	O3—C12—H12B	109.7
C2—C3—H3B	109.5	C13—C12—H12B	109.7
C4—C3—H3B	109.5	H12A—C12—H12B	108.2
H3A—C3—H3B	108.1		
			
C5—C6—C1—N1	171.81 (16)	C6—C7—C14—C15	−120.58 (18)
C7—C6—C1—N1	−5.1 (3)	C8—C7—C14—C15	115.10 (18)
C5—C6—C1—C2	−3.6 (3)	C6—C7—C14—C19	59.5 (2)
C7—C6—C1—C2	179.45 (17)	C8—C7—C14—C19	−64.8 (2)
C9—N1—C1—C6	−14.9 (3)	C20—O5—C18—C19	−4.4 (3)
C9—N1—C1—C2	160.86 (18)	C20—O5—C18—C17	174.56 (19)
C11—C8—C9—N1	−174.65 (17)	C14—C19—C18—O5	178.62 (19)
C7—C8—C9—N1	5.4 (3)	C14—C19—C18—C17	−0.3 (3)
C11—C8—C9—C10	2.4 (3)	C22—O6—C17—C16	91.5 (3)
C7—C8—C9—C10	−177.55 (17)	C22—O6—C17—C18	−90.4 (3)
C1—N1—C9—C8	14.7 (3)	O4—C16—C17—O6	−3.2 (3)
C1—N1—C9—C10	−162.71 (17)	C15—C16—C17—O6	177.59 (18)
C1—C6—C5—O1	−173.80 (19)	O4—C16—C17—C18	178.77 (17)
C7—C6—C5—O1	3.2 (3)	C15—C16—C17—C18	−0.5 (3)
C1—C6—C5—C4	4.2 (3)	O5—C18—C17—O6	3.4 (3)
C7—C6—C5—C4	−178.84 (18)	C19—C18—C17—O6	−177.62 (18)
C21—O4—C16—C15	−2.2 (3)	O5—C18—C17—C16	−178.56 (18)
C21—O4—C16—C17	178.60 (18)	C19—C18—C17—C16	0.5 (3)
C14—C15—C16—O4	−178.84 (17)	C12—O3—C11—O2	0.6 (3)
C14—C15—C16—C17	0.3 (3)	C12—O3—C11—C8	−178.82 (19)
C1—C6—C7—C14	−103.04 (19)	C9—C8—C11—O2	−12.8 (3)
C5—C6—C7—C14	80.0 (2)	C7—C8—C11—O2	167.2 (2)
C1—C6—C7—C8	21.8 (2)	C9—C8—C11—O3	166.62 (17)
C5—C6—C7—C8	−155.10 (16)	C7—C8—C11—O3	−13.4 (2)
C9—C8—C7—C6	−22.0 (2)	C6—C1—C2—C3	−24.4 (3)
C11—C8—C7—C6	158.08 (16)	N1—C1—C2—C3	160.04 (18)
C9—C8—C7—C14	103.36 (19)	C4—C3—C2—C1	49.9 (3)
C11—C8—C7—C14	−76.6 (2)	O1—C5—C4—C3	−158.6 (2)
C16—C15—C14—C19	−0.2 (3)	C6—C5—C4—C3	23.5 (3)
C16—C15—C14—C7	179.89 (16)	C2—C3—C4—C5	−50.4 (3)
C18—C19—C14—C15	0.1 (3)	C11—O3—C12—C13	−164.3 (2)
C18—C19—C14—C7	−179.92 (17)		

### Hydrogen-bond geometry (Å, °)

**Table:**

*D*—H···*A*	*D*—H	H···*A*	*D*···*A*	*D*—H···*A*
C7—H7···O3	0.98	2.36	2.720 (2)	101
N1—H1N···O1^i^	0.83 (3)	2.21 (3)	2.995 (2)	160 (3)
C2—H2B···O4^ii^	0.97	2.55	3.340 (3)	138
C10—H10B···O1^i^	0.96	2.59	3.429 (3)	146

Symmetry codes: (i) *x*−1, *y*, *z*; (ii) *x*−1, *y*−1, *z*.
